# B淋巴细胞肿瘤患者接受CAR-T细胞治疗后巨细胞病毒再激活及其影响因素

**DOI:** 10.3760/cma.j.cn121090-20240703-00249

**Published:** 2024-11

**Authors:** 子豪 王, 凌浩 李, 胜利 薛, 子玲 朱, 杰 徐, 天宇 陆, 荧 王, 惠英 仇, 悦 韩, 苏宁 陈, 晓文 唐, 正明 金, 彩霞 李, 爱宁 孙, 德沛 吴

**Affiliations:** 1 苏州大学附属第一医院血液科，江苏省血液研究所，国家血液系统疾病临床医学研究中心，苏州 215006 The First Affiliated Hospital of Soochow University, National Clinical Research Center for Hematologic Diseases, Jiangsu Institute of Hematology, Suzhou 215006, China; 2 苏州大学附属第一医院检验科，苏州 215006 Department of Laboratory Medicine, the First Affiliated Hospital of Soochow University, Suzhou 215006, China

**Keywords:** 嵌合抗原受体, 巨细胞病毒, 白血病，B细胞, 淋巴瘤，B细胞, Receptors, chimeric antigen, Cytomegalovirus, Leukemia, B-cell, Lymphoma, B-cell

## Abstract

**目的:**

分析接受嵌合抗原受体T（CAR-T）细胞治疗的B淋巴细胞肿瘤患者巨细胞病毒（CMV）再激活情况及其影响因素。

**方法:**

回顾性分析2021年1月至2023年12月于苏州大学附属第一医院接受CAR-T细胞治疗后100 d内进行2次及以上CMV DNA检测和（或）病原体宏基因组测序的B淋巴细胞肿瘤患者的临床资料，比较CMV再激活组和未激活组的临床特征，通过卡方检验和非参数秩和检验分析影响CMV再激活的因素，通过Logistic回归进行危险因素分析。

**结果:**

共纳入86例患者，其中18例（20.9％）发生了CMV再激活，再激活的中位时间为治疗后20（1～95）d，均为CMV血症，未观察到CMV病的发生。7例患者CMV自然转阴，11例患者抗病毒治疗升级为更昔洛韦、膦甲酸钠等一线药物后CMV转为潜伏状态。CAR-T细胞治疗前抗肿瘤治疗疗程数≥6、CAR-T细胞治疗前2年内行allo-HSCT、治疗前肿瘤未缓解、使用大剂量糖皮质激素和（或）托珠单抗与CMV再激活相关，其中治疗前2年内行allo-HSCT和使用大剂量糖皮质激素和（或）托珠单抗治疗是CMV再激活的独立危险因素。

**结论:**

接受CAR-T细胞治疗的B淋巴细胞肿瘤患者存在CMV再激活风险，特别是治疗前2年内行allo-HSCT和接受大剂量糖皮质激素和（或）托珠单抗治疗的患者。

## Abstract (en)

**Objective:**

This study aimed to analyze cytomegalovirus（CMV）reactivation and its influencing factors in patients with B-lymphocyte malignancy who received chimeric antigen receptor T（CAR-T）cell therapy.

**Methods:**

This study retrospectively reviewed patients with B-lymphocyte malignancy who received CAR-T cell therapy in the First Affiliated Hospital of Soochow University from January 2021 to December 2023. The data of patients who underwent CMV-DNA detection and/or pathogen metagenomic sequencing twice or more within 100 days after CAR-T cell therapy were analyzed. The clinical characteristics of the CMV reactivation and non-activation groups were compared. The factors related to CMV reactivation were analyzed with the Chi-square test and nonparametric rank sum test, and the risk factors were examined with Logistic regression.

**Results:**

This study included 86 patients, among whom 18（20.9％）had CMV reactivation, and the median time of reactivation was 20（1–95）days. All of the 18 patients had CMV viremia, and no CMV disease was observed. Seven patients turned to the latent state after continuing acyclovir antiviral therapy, and 11 patients returned to the latent state after upgrading the antiviral therapy to first-line drugs, including ganciclovir and foscarnet sodium. Six or more courses of anti-tumor treatment before CAR-T cell therapy, allogeneic hematopoietic stem cell transplantation within 2 years before CAR-T cell therapy, non-remission before treatment, and the use of high-dose glucocorticoids and/or tocilizumab were related to CMV reactivation, among which allogeneic hematopoietic stem cell transplantation within 2 years pre-treatment and the use of high-dose glucocorticoids and/or tocilizumab treatment were independent risk factors for CMV reactivation.

**Conclusion:**

Patients with B-lymphocyte malignancy who received CAR-T cell therapy have the risk of CMV reactivation, especially for those who received allogeneic hematopoietic stem cell transplantation within 2 years pre-treatment and those who received high-dose glucocorticoids and/or tocilizumab treatment.

巨细胞病毒（CMV）是一种属于乙型疱疹病毒亚科的双链DNA病毒，初次感染后长期潜伏于宿主体内，免疫功能低下时再激活复制而引发相应症状。我国人群中CMV潜伏感染率超过97％[1]，接受异基因造血干细胞移植（allo-HSCT）的患者CMV再激活发生比例高，如引发CMV病则严重危及患者生命，是血液科医师重点关注的病毒感染之一。嵌合抗原受体T（CAR-T）细胞疗法显著提高复发难治性B淋巴细胞肿瘤的总体缓解率和生存率[2]，成为近年来迅速崛起的一种新兴免疫治疗方法，患者接受CAR-T细胞治疗后发生感染风险的临床经验有限。本文回顾性分析接受CAR-T细胞治疗的B淋巴细胞肿瘤患者CMV再激活情况及其影响因素。

## 病例与方法

1. 病例：本研究回顾性分析2021年1月至2023年12月在苏州大学附属第一医院接受CAR-T细胞治疗的B淋巴细胞肿瘤患者，选取回输后100 d内进行≥2次CMV-DNA检测和（或）病原体宏基因组测序的病例作为研究对象，随访至CAR-T细胞治疗后100 d，收集患者的临床特征、CAR-T细胞治疗情况、CMV检测结果等。

2. CAR-T细胞治疗管理：CAR-T细胞产品包括阿基仑赛注射液和临床试验产品（NCT03196830、NCT05470777、NCT03984968、NCT03614858、NCT05691153、NCT05679687）。治疗前进行清除淋巴细胞预处理，治疗后常见的并发症细胞因子释放综合征（CRS）根据中国专家共识进行诊断和分级[3]，对于发生CRS的患者，参照指南使用托珠单抗和（或）糖皮质激素进行治疗。

3. CMV感染的监测及治疗：CMV潜伏、再激活及感染参照《异基因造血干细胞移植患者巨细胞病毒感染管理中国专家共识（2022年版）》[4]进行定义：①CMV潜伏：外周血标本中CMV IgG阳性但未检出病毒核酸；②CMV再激活：内源性潜伏CMV复制增多导致感染；③CMV血症：在外周血标本中检出病毒核酸；④CMV病：有临床症状和体征的CMV感染；⑤难治性CMV感染：接受合理的抗CMV治疗2周后，病毒载量维持不变或上升，CMV病的症状和体征无改善或持续进展。

所有患者治疗前通过酶联免疫吸附法进行CMV IgG检测以确认是否存在CMV潜伏状态。治疗后通过实时定量PCR（RQ-PCR）和（或）病原体宏基因组测序检测患者外周血CMV核酸水平。以RQ-PCR结果大于500拷贝/ml和（或）病原体宏基因组测序检出CMV基因序列作为病毒核酸检出标准。

所有患者治疗前开始长期口服阿昔洛韦预防疱疹病毒感染，CMV治疗包括更昔洛韦、膦甲酸钠和CMV人免疫球蛋白等。

4. 随访：采用查阅门诊/住院电子病历和电话进行随访，随访截止日期为2024年3月31日。

5. 统计学处理：采用SPSS 26.0软件进行统计学分析。连续变量用中位数（范围）描述，分类变量用例数（百分比）描述。单因素分析时，连续变量用非参数秩和检验，分类变量用卡方检验。将单因素分析中*P*<0.05的变量纳入多因素分析。双侧*P*<0.05为差异有统计学意义。

## 结果

1. 临床特征：86例患者中，男45例（52.3％），女41例（47.7％），中位年龄42（14～78）岁。59例（68.6％）为B细胞急性淋巴细胞白血病（ALL），27例（31.4％）为非霍奇金淋巴瘤（NHL）（弥漫大B细胞淋巴瘤22例，伯基特淋巴瘤3例，套细胞淋巴瘤2例）。38例（44.2％）患者接受CD19单靶点CAR-T细胞治疗，48例（55.8％）患者接受CD19和CD22双靶点CAR-T细胞治疗。

对比CMV再激活组和未激活组患者的临床特征，两组既往抗肿瘤治疗疗程数（*P*＝0.046）及CAR-T细胞输注剂量（*P*＝0.026）的差异有统计学意义，两组患者性别、年龄、诊断、ECOG评分、CAR-T细胞靶点、CRS等级、既往是否单抗治疗、既往是否行allo-HSCT及桥接allo-HSCT的差异均无统计学意义（*P*值均>0.05）（表1）。

**表1:** CAR-T细胞治疗后CMV再激活和未激活的B淋巴细胞肿瘤患者临床特征比较

临床特征	CMV再激活（18例）	CMV未激活（68例）	*χ*^2^值/*Z*值	*P*值
性别［例（％）］			0.095	0.758
男	10（55.6）	35（51.5）		
女	8（44.4）	33（48.5）		
年龄［岁，*M*（范围）］	46（22～78）	41.5（14～66）	−1.476	0.140
诊断［例（％）］			0.138	0.710
ALL	13（72.2）	46（67.6）		
NHL	5（27.8）	22（32.4）		
ECOG评分［例（％）］			−0.258	0.797
0	0（0）	1（1.5）		
1	15（83.3）	56（82.4）		
2	2（11.1）	11（16.2）		
3	1（5.6）	0（0）		
CAR-T细胞靶点［例（％）］			2.644	0.104
CD19	11（61.1）	27（39.7）		
CD19、CD22	7（39.9）	41（60.3）		
CAR-T细胞输注剂量［×10^7^/kg，*M*（范围）］	0.3（0.1～2.0）	1.0（0.1～2.0）	−2.229	0.026
CRS等级［例（％）］			1.417	0.234
0～1级	11（61.1）	50（73.5）		
≥2级	7（38.9）	18（26.5）		
既往抗肿瘤治疗疗程数［*M*（范围）］	6（2～26）	4（2～29）	−2.002	0.046
既往单抗治疗［例（％）］			2.290	0.130
有	9（50.0）	21（30.9）		
无	9（50.0）	47（69.1）		
既往行allo-HSCT［例（％）］			1.733	0.188
有	5（27.8）	8（13.3）		
无	13（72.2）	60（86.7）		
桥接allo-HSCT［例（％）］			2.293	0.130
有	3（16.7）	24（35.3）		
无	15（83.3）	44（64.7）		

**注** CAR-T细胞：嵌合抗原受体T细胞；CMV：巨细胞病毒；ALL：急性淋巴细胞白血病；NHL：非霍奇金淋巴瘤；CRS：细胞因子释放综合征；allo-HSCT：异基因造血干细胞移植

2. CMV再激活情况及治疗转归：86例B淋巴细胞肿瘤患者接受CAR-T细胞治疗前均为CMV潜伏状态，其中18例（20.9％）在治疗后100 d内发生了CMV再激活，中位发生时间为治疗后20（1～95）d。

所有发生CMV再激活的患者均表现为CMV血症，无CMV病发生，经治疗均在2周内得到控制，CMV DNA水平降至检测阈值以下。其中7例（38.9％）患者继续接受阿昔洛韦治疗，未更改或增加药物。另外11例（61.1％）患者加用其他治疗：3例应用阿昔洛韦、更昔洛韦，3例应用阿昔洛韦、膦甲酸钠，2例应用阿昔洛韦、膦甲酸钠、CMV人免疫球蛋白，1例应用阿昔洛韦、更昔洛韦、CMV人免疫球蛋白，1例应用阿昔洛韦、CMV人免疫球蛋白、来特莫韦，1例应用阿昔洛韦、更昔洛韦、膦甲酸钠、缬更昔洛韦。

3. 危险因素分析：单因素分析显示，既往抗肿瘤治疗疗程数≥6（*P*＝0.040）、治疗前肿瘤未缓解（*P*＝0.034）、治疗前2年内行allo-HSCT（*P*＝0.047）、治疗后使用大剂量糖皮质激素和（或）托珠单抗（*P*＝0.023）与CMV再激活相关（表2）。对单因素分析中*P*<0.05的变量进行多因素分析，CAR-T细胞治疗前2年内行allo-HSCT（*OR*＝18.012，95％*CI* 2.340～138.649，*P*＝0.005）和CAR-T细胞治疗后使用大剂量糖皮质激素和（或）托珠单抗（*OR*＝5.836，95％*CI* 1.438～23.691，*P*＝0.014）是CMV再激活的独立危险因素（表2）。

**表2:** B淋巴细胞肿瘤患者CAR-T细胞治疗后CMV再激活的影响因素

因素	单因素分析		多因素分析	
	*P*值	*OR*（95％*CI*）	*P*值	*OR*（95％*CI*）
年龄（>55岁，≤55岁）	0.283	1.790（0.619～5.181）		
性别（男，女）	0.758	1.179（0.415～3.349）		
诊断（ALL，NHL）	0.710	1.243（0.394～3.926）		
既往抗肿瘤治疗疗程数（≥6，<6）	0.040	3.075（1.052～8.986）	0.150	2.566（0.711～9.259）
既往单抗治疗（有，无）	0.135	2.238（0.777～6.444）		
治疗前肿瘤未缓解（是，否）	0.034	0.269（0.080～0.903）	0.130	0.344（0.087～1.367）
治疗前2年内行allo-HSCT（是，否）	0.047	4.571（1.018～20.523）	0.005	18.012（2.340～138.649）
CAR-T细胞靶点（CD19，CD19、CD22）	0.109	2.386（0.823～6.921）		
CRS级别（≥2级，0~1级）	0.239	1.944（0.643～5.876）		
使用大剂量糖皮质激素和（或）托珠单抗（是，否）	0.023	4.286（1.223～15.022）	0.014	5.836（1.438～23.691）
桥接allo-HSCT（是，否）	0.141	0.367（0.096～1.394）		

**注** CAR-T细胞：嵌合抗原受体T细胞；CMV：巨细胞病毒；ALL：急性淋巴细胞白血病；NHL：非霍奇金淋巴瘤；allo-HSCT：异基因造血干细胞移植；CRS：细胞因子释放综合征

## 讨论

CMV再激活与患者的免疫功能低下密切相关，接受免疫抑制治疗的肿瘤患者有显著的再激活发生率[5]，在接受allo-HSCT的血液肿瘤患者中，CMV再激活的中位发生率高达37％，是导致非复发死亡的重要原因[6]。作为目前接受CAR-T细胞治疗的主要疾病类型，B淋巴细胞肿瘤存在B淋巴细胞功能缺陷、淋巴细胞清除预处理、持久的B淋巴细胞再生障碍及低丙种球蛋白血症等免疫功能低下情况[7]–[8]，有必要研究此类患者的CMV再激活情况。

近年来CAR-T细胞治疗后CMV再激活已引起人们的重视，其在CAR-T细胞治疗后的病毒感染中高居榜首[9]，发生率为17％～56.4％，多在40％以上[9]–[13]。本研究显示，B淋巴细胞肿瘤患者接受CAR-T细胞治疗后CMV再激活发生率为20.9％。各报道中CMV再激活发生率不同可能与不同研究的患者CMV潜伏状态、CMV监测频率、预防性抗病毒治疗、疾病类型等存在差异相关。本研究中CMV再激活的中位时间为治疗后20 d，与既往报道的CMV再激活时间集中在CAR-T细胞治疗后2～6周一致[13]。结合本研究和其他研究的结果[9]–[13]，CAR-T细胞治疗后CMV再激活总体可控，CMV病的发生少见，部分具有自限性，部分经抗病毒治疗转为潜伏状态，但也有死亡病例。

本研究显示，CAR-T细胞治疗前2年内行allo-HSCT和CAR-T细胞治疗后使用大剂量糖皮质激素和（或）托珠单抗是CMV再激活的独立危险因素。CAR-T细胞治疗常发生CRS，通常使用糖皮质激素、托珠单抗等进行治疗。其他研究也证实，应用糖皮质激素与CMV再激活相关[10],[13]，而托珠单抗在控制CRS的同时也会损害患者的免疫功能[14]，其导致CMV再激活的具体机制值得进一步研究。allo-HSCT后，免疫系统各组成部分的重建进程不同[15]。固有免疫首先重建，中性粒细胞和单核细胞通常在移植后1周至1个月可恢复正常；适应性免疫恢复较慢，尤其是CD4^+^T细胞，在移植后1～2年内才能完成重建[16]–[17]。CD4^+^T淋巴细胞在CMV特异性免疫中发挥重要作用[18]，CMV再激活发生率随CD4^+^T细胞数量的下降而升高[19]–[20]。上述研究为CAR-T细胞治疗前2年内行allo-HSCT的B淋巴细胞肿瘤患者具有更高的CMV再激活风险提供了理论支持。

总而言之，接受CAR-T细胞治疗的B淋巴细胞肿瘤患者存在较高的CMV再激活风险，多数CMV血症总体治疗反应良好。CAR-T细胞治疗前2年内行allo-HSCT、CAR-T细胞治疗后使用大剂量糖皮质激素和（或）托珠单抗是CMV再激活的独立危险因素。建议接受CAR-T细胞治疗后的B淋巴细胞肿瘤患者加强CMV监测，特别是具有以上2项危险因素的患者，以便及时发现和处理CMV感染，及时应用抗病毒药物预防CMV再激活发生。
